# Heuristic algorithms for the minmax regret flow-shop problem with interval processing times

**DOI:** 10.1007/s10100-017-0485-8

**Published:** 2017-07-29

**Authors:** Michał Ćwik, Jerzy Józefczyk

**Affiliations:** Faculty of Computer Science and Management, Wroclaw University of Science and Technology, Wybrzeze Wyspianskiego 27, 50-370 Wrocław, Poland

**Keywords:** Flow-shop, Interval processing times, Minmax regret, Heuristic algorithms, Computational experiments

## Abstract

An uncertain version of the permutation flow-shop with unlimited buffers and the makespan as a criterion is considered. The investigated parametric uncertainty is represented by given interval-valued processing times. The maximum regret is used for the evaluation of uncertainty. Consequently, the minmax regret discrete optimization problem is solved. Due to its high complexity, two relaxations are applied to simplify the optimization procedure. First of all, a greedy procedure is used for calculating the criterion’s value, as such calculation is NP-hard problem itself. Moreover, the lower bound is used instead of solving the internal deterministic flow-shop. The constructive heuristic algorithm is applied for the relaxed optimization problem. The algorithm is compared with previously elaborated other heuristic algorithms basing on the evolutionary and the middle interval approaches. The conducted computational experiments showed the advantage of the constructive heuristic algorithm with regards to both the criterion and the time of computations. The Wilcoxon paired-rank statistical test confirmed this conclusion.

## Introduction

The paper develops a minmax regret approach as a special case of uncertain (non-deterministic) discrete optimization with reference to selected flow-shop problem when processing times are given in the form of intervals. The contribution and significance of results can be considered both from the minmax regret discrete optimization and from flow-shop problems points of view. In general, dealing with any non-deterministic discrete optimization has to be focused on three fundamental issues: the representation of uncertainty, the evaluation of feasible solutions (optimization variables) and the development of solution algorithms [see Kouvelis and Yu (1997), Kasperski (2008), and Józefczyk and Ćwik (2016) for more detailed discussion]. A particular fusion of selected options from all the issues constitutes a problem investigated in the paper.

Namely, the interval representation of problem’s uncertain parameters is assumed; the parametric uncertainty is only considered in the paper. Such a choice, which abandons other more popular representations like probabilistic, possibilistic, fuzzy, uncertain variables-based, refers to prospective real-world applications when empirical data, as well as experts’ knowledge, are not available, e.g. for one-off processes. A detailed description of different approaches for the representation of uncertainty and their applications can be found e.g. in: Hirshleifer and Riley (1979), Bubnicki (2004), Aissi et al. (2009), Klir (2006), Aayyub and Klir (2006), and Liu (2010). It is worth mentioning that the probabilistic representation is the most popular. Then, it is assumed that a parameter’s value is the realization of a random variable characterized by the probability distribution function or the density function being the probabilistic representations of an uncertain parameter. It is the objective representation of uncertainty as it is potentially possible to estimate the probability distribution using real-world data on an uncertain parameter. Such possibility makes this representation very sound admittedly, but it is connected with its weakness when real-world data on the parameter do not exist, or they are not available, and, in consequence, it is impossible to obtain reliable probability distribution. The lack of the real-world data can be replaced by an expert who is assumed as a source of knowledge on uncertain parameters. Such an approach is proposed in fuzzy based representations and its derivative versions. The degree of truth as a number from the interval [0, 1] is expressed by an expert that a parameter takes a given value. Expert’s opinion for all possible values of a parameter is called the membership function. This representation is the subjective one as it reflects an individual opinion on a value of a parameter, and it does not have to be connected with parameter’s real-world values. In consequence, the final result of the decision making, in general, and the determination of a schedule, in particular, strongly depends on the credibility (quality) of an expert. The case considered by many researchers omitting the drawbacks of both previously mentioned representations assumes that set of feasible values is the only information on an uncertain parameter. The interval representation discussed in this paper is an important example of this case.

The second issue deals with a criterion. The substantiation (aggregation, determinization) of parameters’ uncertainty by an adequate operator is an indispensable condition for having a unique solution for an uncertain problem. The majority of investigated uncertain discrete optimization cases are based on such an approach, e.g. Yager (1988), and Kasperski and Zieliński (2013). Among many possibilities, the minmax regret approach has been selected and applied in the paper. It is based on the notion ‘regret’ introduced in Savage (1951) and then developed by other authors for discrete optimization, e.g. Kouvelis and Yu (1997), Aissi et al. (2009), Mulvey et al. (1994), and Kasperski (2008). The direction launched by Kouvelis and Yu is the basis for further considerations. The regret is a function founded on a criterion for a deterministic counterpart of the considered uncertain optimization. Its value, which can be calculated for every instance of optimization variables as well as for every realization (scenario) of uncertain parameters, evaluates the loss caused by a lack of knowledge of the uncertain parameters’ values. The substantiation by means of the operator ‘maximum’ applied for the regret implies the minmax regret uncertain discrete optimization. The resulted deterministic criterion evaluates optimization variables giving the assessment of regret the worst with respect to all possible scenarios of uncertain parameters. It is necessary to point out that such an evaluation when the robust paradigm, as well as the unique solution of the uncertain problem, are required should be considered as the selected approach taken from a variety of possibilities reported in the literature. Let us briefly mention other selected options. The way of evaluation strongly depends on the form of parameter uncertainty representation. Essentially, criteria relevant for deterministic problems or their functions, like the regret in this paper, are the basis for evaluation of the problems’ uncertain counterparts. They can be further transformed using a selected aggregation operator with ‘maximum’, ‘minimum’ or ‘average’ as the most popular ones. The expected value as an example of ‘average’ operator is very often used for the probabilistic representation, e.g. Sotskov and Werner (2014), and Pinedo and Schrage (1982). Uncertain problems with fuzzy representation are usually considered in the setting of possibility theory where the evaluation of solutions is expressed by the degrees of possibility and necessity, e.g. Kasperski (2008), and Słowinski and Hapke (1999). Irrespective of the form of parameter uncertainty representation, two general cases can be distinguished: the determination of a unique solution or a set of solutions. The former case is considered in this paper. It is also a typical for probabilistic or fuzzy representations when we search for solutions minimizing the expected value or the possibility-based criterion, respectively. The latter case usually imposes weaker requirements on the criteria, and, as a consequence, enables us to have a set of feasible solutions instead of a single one. Three measures adequate for the determination of sets of feasible solutions and suitable for the interval representation can be mentioned as the example: (*b*, *w*)-robustness (Roy 2010), *p*-robustness (Kouvelis et al. 1992), and lexicographic $\alpha$-robustness (Kalai et al. 2012). See also the survey (Goerigk and Schöbel 2016) for more details. The outcome in the form of a set of feasible solutions conforming given a priori requirements is reasonable due to the existence of uncertainty in the values of parameters. The rule ‘less certain a priori information on parameters less precise solution’ is sound. However, the existence and the form of the final solution substantially depend on the numerical values of parameters, *b*, *w*, *p*, and $\alpha$. The idea of searching for the set of solutions rather than for an individual solution has also been employed by so-called stability approach (Sotskov and Werner 2014; Lai et al. 1997). It makes possible to derive a minimal dominant set of solutions such that at least one of them is optimal for any fixed realization of uncertain parameters. The method uses a stability analysis to assess such changes of the parameters’ values which do not lose the optimality in solutions. This method has also been applied for two-machine flow-shop with interval processing times and the makespan as a criterion (Allahverdi et al. 2003; Matsveichuk et al. 2009; Ng et al. 2009). The authors additionally propose for this application of the stability approach two phases in the scheduling process: the off-line phase for the schedule planning and the on-line phase when the schedule is executed taking into account current additional information on the previously uncertain processing times’ values.

The third mentioned issue regarding solution algorithms for such optimization problems is the most important and challenging. Unfortunately, apart from single cases, the majority of minmax regret discrete optimization problems are at least NP-hard. Their time complexity strongly depends on this property for deterministic counterparts. Some interesting results have been attained for problems with easy deterministic counterparts where polynomial algorithms are known. Then, despite the NP-hardness of uncertain counterparts, it was able to propose approximate algorithms. For example, it concerns general problems, e.g. Conde (2010) as well as particular problems like: shortest path (Aissi et al. 2005b), assignment (Aissi et al. 2005) or elementary task scheduling e.g. Kasperski and Zieliński (2008), and Józefczyk and Siepak (2014). Unfortunately, no approximation algorithms can exist for uncertain problems with at least NP-hard deterministic counterparts. Then, heuristic algorithms are a reasonable way to have solutions in the acceptable time. It is essential for practical usage of the considered uncertain discrete optimization. The development of such algorithms is an important research challenge. First attempts of this research direction can be found in Kasperski et al. (2012), and Averbakh and Pereira (2011) for minimum spanning tree and assignment problems, respectively. The application of scatter search based methods for minmax regret task scheduling is presented in Siepak (2013), Józefczyk and Siepak (2013), and Józefczyk and Siepak (2014). Following these works, the paper contributes to heuristic methods as tools for solving minmax regret optimization problems with the interval parametric uncertainty.

In the paper, the considerations are confined to selected flow-shop problem, and the most important presented results regard this area. The permutation version of flow-shop with unlimited buffers and the makespan as a criterion is considered, e.g. Pinedo (2008), Błażewicz et al. (2007), and Chakrraborty (2009). It is the important task scheduling problem with many important applications, in particular in management and manufacturing, e.g. Hajba and Horvath (2015). The deterministic version of flow-shop for two machines is easy, and the polynomial algorithm exists (Garey et al. 1976). For a bigger number of machines, the problem becomes NP-hard. The minmax regret with interval uncertainty version of the considered flow-shop was also investigated for some cases. Namely, the proof of its NP-hardness is given in Daniels et al. (2000), the problem with two jobs is studied by Averbakh (2006), the problem with two machines and discrete scenarios is discussed in Kasperski et al. (2012) where 2-approximation algorithm is also given, while the 2-machine problem is dealt with by Daniels et al. (2000). All these works show that the interval minmax regret flow-shop is at least NP-hard even for simple special cases e.g. for the number of machines limited to two.

The continuation of previous preliminary studies given in Ćwik and Józefczyk (2015) is proposed where the evolutionary algorithm has been presented for only three machines and the exact evaluation of the maximum regret as the criterion. Such a way of the criterion evaluation turned out impossible for more machines due to the time complexity. Therefore, another approach for the calculation of maximum regret has been applied and firstly announced in the conference presentation (Józefczyk and Ćwik 2016). The main contribution of this paper consists in the elaboration of a new constructive algorithm useful also for more than three machines, which outperforms the previously developed evolutionary algorithm. To our best knowledge, there are no other works in the literature on heuristic algorithms for the minmax regret flow-shop with interval parameters. The paper attempts to fill this research gap studying selected heuristic approaches for the flow-shop and referring to similar works for other minmax regret problems with interval parameters.

The reminder of this paper is organized as follows. Section 2 gives the problem formulation which then is analyzed and relaxed for its simpler version. Three heuristic algorithms are presented in Sect. 3: first of all, a constructive algorithm referring to known NEH heuristic, an evolutionary algorithm, and a simple middle interval-based algorithm. Described more briefly two latter algorithms serve as a comparison for the former one. Section 4 is devoted to the presentation of results of the computational experiments which affirmed the applicability of the constructive and evolutionary algorithms for real-world instances with the significant advantage of the first one. Section 5 contains conclusions and directions for further research.

## Problem statement

In this section, we provide formal definitions for both deterministic and uncertain permutation flow shop problems.

### Deterministic case

Let us consider a set $${\mathbf{J}}=\{J_1 ,J_2 ,\ldots ,J_j ,\ldots ,J_n \}$$ of *n* jobs which need to be scheduled for processing on *m* machines from a set $${\mathbf{M}}=\{M_1 ,M_2 ,\ldots ,M_i ,\ldots ,M_m \}$$. Each job $$J_j =(O_{1j} ,O_{2j} ,\ldots ,O_{ij} ,\ldots ,O_{mj})$$ consists of *m* operations which must be processed sequentially on all machines in the order indicated by the machines’ indexes. No job can be processed by more than one machine at the same time. Similarly, no machine is allowed to process more than one job at a given moment of time. The existence of unlimited buffers is assumed that enables every machine to process the next job just after processing of the previous one. If the next machine is unavailable, the job can wait for processing in the buffer without occupying any of the machines. The buffers can contain any number of jobs. Moreover, the permutation version of the problem is investigated which means that all machines have to process the jobs in the same order. Then the schedule being the solution to the problem can be represented by a permutation $$\pi =(\pi _j )_{j=\overline{1,n} } \in \varvec{\Pi } $$ where $$\pi _j \in \{1,2,\ldots ,n\}$$ is the index of a job processed as the *j*th in turn, and $$\varvec{\Pi } $$ is the set of all *n*! feasible permutations, i.e. $$\varvec{\Pi } =\{\pi :\pi _j \ne \pi _k ,j,k\in \{1,2,\ldots ,n\},j\ne k\}$$. Processing times $$p_{ij} $$ of operations $$O_{ij} $$ form the matrix $$p=[p_{ij} ]_{\mathop {i=\overline{1,m}}\limits _{j=\overline{1,n}}}$$. The makespan $$C_{\max } (\pi ,p)$$, as the completion time of the last operation executed by the machine $$M_m$$, serves as a criterion. It can be recursively calculated [see e.g. in Pinedo (2008), and Ćwik and Józefczyk (2015) for details]. The optimal schedule $${\pi }'$$ is sought, i.e. $$\mathop {\min }\nolimits _{\pi \in \varvec{\Pi } } C_{\max } (\pi ,p)=C_{\max } ({\pi }',p)\triangleq {C}'_{\max }(p)$$.

### Uncertain case

It is assumed for the considered uncertain version that every processing time belongs to the closed interval of known and given bounds, i.e.:

1 $$\begin{aligned} p_{ij} \in \left[ \underline{p}_{ij} ,\;\bar{{p}}_{ij} \right] ,\;\underline{p}_{ij} \le \bar{{p}}_{ij} \end{aligned}$$

unlike the deterministic version when $p_{ij}$ are crisp values. The Cartesian product of all *mn* intervals constitutes a set **P** of all possible scenarios. A scenario is a unique set of processing times of all operations which can be considered as an instance of the deterministic problem, i.e. the matrix *p*:

2 $$\begin{aligned} p\in \mathbf{P}=\left[ \underline{p}_{11} ,\;\bar{{p}}_{11} \right] \times \cdots \times \left[ \underline{p}_{mn} ,\bar{{p}}_{mn}\right] . \end{aligned}$$

The minmax regret approach is applied to evaluate the interval uncertainty (Kouvelis and Yu 1997).

The regret $C_{\max } (\pi ,p)-{C}'_{\max } (p)$ is defined for every schedule $\pi$ and scenario *p*. The scenario $p^{\pi }$ maximizing the regret for fixed schedule $\pi$ is called a worst-case scenario. The regret associated with $\pi$ and $p^{\pi }$ referred to as maximum regret constitutes the criterion *z* for the interval data counterpart of the permutation flow-shop problem:

3 $$\begin{aligned} z(\pi )=C_{\max } (\pi ,p^{\pi })-{C}'_{\max } (p^{\pi })=\mathop {\max }\limits _{p\in \mathbf{P}} \left[ C_{\max } (\pi ,p)-\mathop {\min }\limits _{\sigma \in \varvec{\Pi } } C_{\max } (\sigma ,p)\right] . \end{aligned}$$

Consequently, the uncertain (interval) minmax regret permutation flow shop problem considered in the paper deals with the minimization of (3) over $\pi \in \varvec{\Pi }$. This problem has been already proven to be NP-hard (Daniels et al. 2000) even for $m=2$. Moreover, there exists 2-approximate algorithm when $m=2$ as shown in Siepak (2013), Obviously, such approximation is not valid for larger values of *m* due to the NP-hardness of the deterministic counterpart. The existence of the approximate algorithm for the uncertain version would induce its validity for the deterministic instance as the special case for all processing times with the same lower and upper bounds of intervals, which is not true unless P $=$ NP.

In fact, the minimization of (3) consists of three nested optimizations (sub-problems) called SP1, SP2, and SP3 (Józefczyk and Ćwik 2016). The sub-problem SP1: $$\mathop {\min }\nolimits _{\sigma \in \varvec{\Pi } } C_{\max } (\sigma ,p)$$ is simply the deterministic flow shop. The next sub-problem SP2: $$\mathop {\max }\nolimits _{p\in \mathbf{P}} [C_{\max } (\pi ,p)-{C}'_{\max } (p)]$$ deals with finding the worst-case scenario $$p^{\pi }$$, and, at the same time, calculating of the value of criterion $$z(\pi )$$ for fixed schedule $$\pi $$. The outer minimization $$\mathop {\min }\nolimits _{\pi \in \varvec{\Pi } } z(\pi )=\mathop {\min }\nolimits _{\pi \in \varvec{\Pi } } [C_{\max } (\pi ,p^{\pi })-{C}'_{\max } (p^{\pi })]$$, i.e. the sub-problem SP3 completes this complex optimization task. All the sub-problems are difficult optimization points. SP1 is NP-hard for $$m>2$$ (Pinedo 2008). Searching for the worst-case scenario in SP2 can be limited to the consideration of the bounds of intervals referred to as extreme-points scenarios, i.e. $$p_{ij}^\pi \in \{\underline{p}_{ij} ,\bar{{p}}_{ij} \}$$. It is the known result for a broad class of minmax regret problems valid also for the investigated case. It is easy to see that both elements of the difference to be maximized $$C_{\max } (\pi ,p)-C^{\prime }_{\max } (p)$$ refer to different feasible solutions. Every such element as the makespan is the sum of some processing times $$p_{ij} $$. To maximize the difference, it is necessary to have the maximum and the minimum value of the first and the second sum, respectively. It is straightforwardly ensured by the extreme-points scenarios. As a consequence, $$2^{nm}$$ possible scenarios remain still as candidates for the worst-case one $$p^{\pi }$$. The minimization in SP3 can be considered as a harder task than the deterministic flow-shop. Indeed, the regret $$C_{\max } (\pi ,p^{\pi })-{C}'_{\max } (p^{\pi })$$ undergoes the minimization with respect to $$\pi \in \varvec{\Pi } $$ in SP3 while the minimization of $$C_{\max } (\pi ,p^{\pi })$$ would be only required for the deterministic case. Obviously, SP3 is also NP-hard for $$m>2$$.

## Heuristic solution algorithms

We have considered during hitherto investigations two algorithms for solving SP3: the application of the evolutionary approach, and the elaboration of a constructive heuristic algorithm referring to the NEH heuristic known for the deterministic case (Enscore et al. 1983). The former one has been presented in Ćwik and Józefczyk (2015) and Józefczyk and Ćwik (2016) for the first time while the latter one together with the improved version of the evolutionary approach is given in this paper. Both algorithms are accompanied with procedures (auxiliary algorithms) responsible for solving SP1 and SP2. All the algorithms are presented in the consecutive sub-sections starting from the heuristic procedure enabling the determination of the worst-scenario in SP2. Additionally, a middle interval heuristic is given which together with the evolutionary algorithm serves for the evaluation of the constructive algorithm.

### Calculation of the worst-case scenario

Firstly, instead of calculating the exact value of $C^{\prime }_{\max } (p)$ as the result of minimization in SP1, the lower bound ${C}'_{\max , \mathrm{LB}} (p)$ is applied which is the maximum sum of a single job processing times:

4 $$\begin{aligned} {C}'_{\max , \mathrm{LB}} (p)=\mathop {\max }\limits _{j=\overline{1,n} } {\sum }_{i=1}^m {p_{ij} } . \end{aligned}$$

The determination of the worst-case scenario $$p^{\pi }$$ can be replaced by a path in a directed acyclic graph constructed using a feasible solution $$\pi $$. It refers to the known representation of selected task scheduling problems in the form of disjunctive graph (Pinedo 2008; Błażewicz et al. 2007). The number of disjunctive graph vertexes is *mn* (each vertex represents a single operation of the problem). Let us assume that the vertices are indexed in the same manner as operations, so vertex $$v_{ij} $$ is associated with operation $$O_{i,\pi _j } $$. The set of arcs is defined by the precedence constraints deduced from the schedule. There exists an edge between all pairs of vertexes that have a form of $$(v_{ij} ,v_{i+1,j} )$$ or $$(v_{ij} ,v_{i,j+1} )$$. From each of $$(n+m-2)!/[(n-1)!(m-1)!]$$ possible paths, a scenario is created by choosing maximum processing times for operations associated with vertexes belonging to the path and minimum processing times for other operations. Although the number of paths is significantly smaller than the number of possible extreme-point scenarios which equals to $$2^{mn}$$, it is too big to check all paths in a reasonable time. Thus, an approximation of $$p^{\pi }$$ is obtained by a heuristic construction of a path in the above-introduced directed graph. The algorithm determines a partial path for each vertex in the directed graph and returns the path determined for the vertex $$v_{mn} $$. Each partial path is created by choosing one of two candidate paths. The candidate paths are generated by adding currently investigated vertex $$v_{ij} $$ to the previously determined paths for vertexes $$v_{i-1,j} $$ and $$v_{i,j-1} $$. Each candidate path is then transformed to a partial scenario with *j* jobs and *i* machines, which is evaluated by the simplified regret function using $${C}'_{\max , \mathrm{LB}} (p)$$ instead of $${C}'_{\max } (p).$$ The path generating of the scenario with a greater value of the regret is chosen as the partial path for the investigated vertex. It can be easily observed that for each pair of vertexes $$v_{i,1} $$ and $$v_{1,j} $$ there exists only one path that ends in them. Therefore, the determination of those paths is the first step of the algorithm. Let us additionally introduce the matrix $$pp=[pp_{ij} ]_{\mathop {i=\overline{1,m}}\nolimits _{j=\overline{1,n}}}$$ which elements contain the partial paths created for each operation. Then, the auxiliary algorithm referred to as Algorithm 1 for creating of the path in directed graph and of the relaxed worst-case scenario $$\tilde{p}^{\pi }$$ can be equivalently presented in the form of following pseudocode.

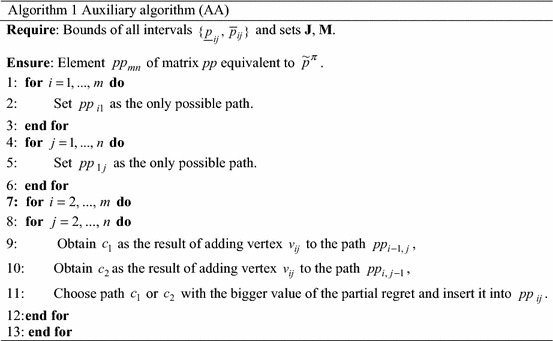



As a consequence, function $z(\pi )$ to be minimized with respect to $\pi$ in the sub-problem SP3 takes the relaxed form

5 $$\begin{aligned} \tilde{z}(\pi )=C_{\max } (\pi ,\tilde{p}^{\pi })-{C}'_{\max , \mathrm{LB}} (\tilde{p}^{\pi }). \end{aligned}$$

This form of $z(\pi )$ is the basis for further considerations. The separate application of both relaxations presented above to function *z* given in (3) results in the following estimations:

6 $$\begin{aligned} C_{\max } (\pi ,\tilde{p}^{\pi })-{C}'_{\max } (\tilde{p}^{\pi })\le z(\pi )\le \mathop {\max }\limits _{p\in \mathbf{P}}\left[ C_{\max } (\pi ,p)-{C}'_{\max , \mathrm{LB}} (p)\right] . \end{aligned}$$

It is worth noting that the relaxations of SP1 and SP2 are opposed. The application of the lower bound in SP1 increases the value of (3):

7 $$\begin{aligned} \mathop {\max }\limits _{p\in \mathbf{P}} \left[ C_{\max } (\pi ,p)-\mathop {\min }\limits _{\sigma \in \varvec{\Pi } } C_{\max } (\sigma ,p)\right] \le \mathop {\max }\limits _{p\in \mathbf{P}} \left[ C_{\max } (\pi ,p)-{C}'_{\max , \mathrm{LB}} (p)\right] . \end{aligned}$$

On the other hand, the greedy calculation of the worst-case scenario in SP2 can decrease the value of (3):

8 $$\begin{aligned} \mathop {\max }\limits _{p\in \mathbf{P}}\left[ C_{\max } (\pi ,p)-C^{\prime }_{\max } (p)\right] \ge C_{\max } (\pi ,\tilde{p}^{\pi })-C^{\prime }_{\max } (\tilde{p}^{\pi }). \end{aligned}$$

In consequence, the relation between $z(\pi )$ and $\tilde{z}(\pi )$ is unclear. However, the bounds on $z(\pi )$ given in (6) are also valid for $\tilde{z}(\pi )$ i.e.:

9 $$\begin{aligned} C_{\max } (\pi ,\tilde{p}^{\pi })-{C}'_{\max } (\tilde{p}^{\pi })\le \tilde{z}(\pi )\le \mathop {\max }\limits _{p\in \mathbf{P}} \left[ C_{\max } (\pi ,p)-{C}'_{\max , \mathrm{LB}} (p)\right] \end{aligned}$$

which results from a simple comparison of both bounds with the right-hand side of (5). The usage of lower bound in SP1 and the approximate solution of SP2 affect conversely on the value of (3) that can be treated as the desirable feature of the proposed heuristic approach.

### Constructive algorithm (CVE)

A heuristic approach proposed in this paper is based on the constructive method of the generation of permutations. For the considered problem, such method consists in the iteratively repeated insertions of jobs into the best positions of the current partial permutation until all jobs are scheduled. The idea refers to the algorithm NEH (Enscore et al. 1983) known as the effective heuristic solution tool for the deterministic flow shop. NEH is composed of two steps which have to be adapted for the uncertain case. In the first step, the middle values of intervals are used to order tasks for the second step. In the second step, the values of maximum regret $\tilde{z}$ are used in order to determine the best position in the partial permutation of the inserted job, unlike NEH where the values of makespan are simply used. The heuristic solution $\pi ^{\mathrm{CVE}}$ as well as $\tilde{z}(\pi ^{\mathrm{CVE}})$ are the results. The pseudocode gives more details of Algorithm 2 denoted also as CVE.

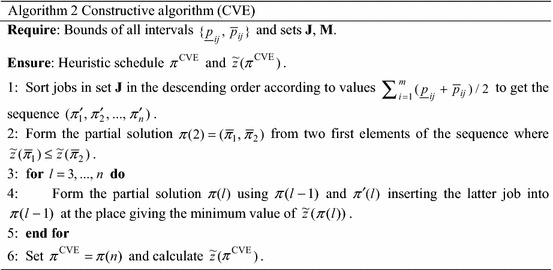



### Other algorithms

#### Middle interval heuristic algorithm (MIH)

The midpoint approach consists in generating a deterministic instance of the problem using middle points of all uncertainty intervals and then in solving the obtained deterministic instance with an exact algorithm. For many minmax regret problems, this algorithm has been proven to be 2-approximate, e.g. Aissi et al. (2009), and Conde (2010). However, in this case, there is no polynomial exact algorithm available due to NP-hardness of the deterministic version of the problem. The NEH heuristic has been proven to be an effective method of solving permutation flow shop problems. Therefore it is used in the second step of Algorithm 3 which generates a heuristic solution denoted as $\pi ^{\mathrm{MIH}}$.

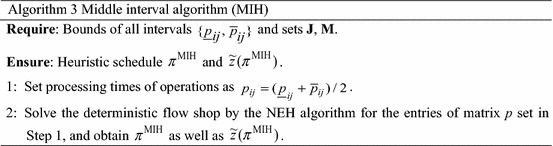



#### Evolutionary algorithm (*EVO*)

The used evolutionary algorithm can be briefly presented as the following pseudocode.

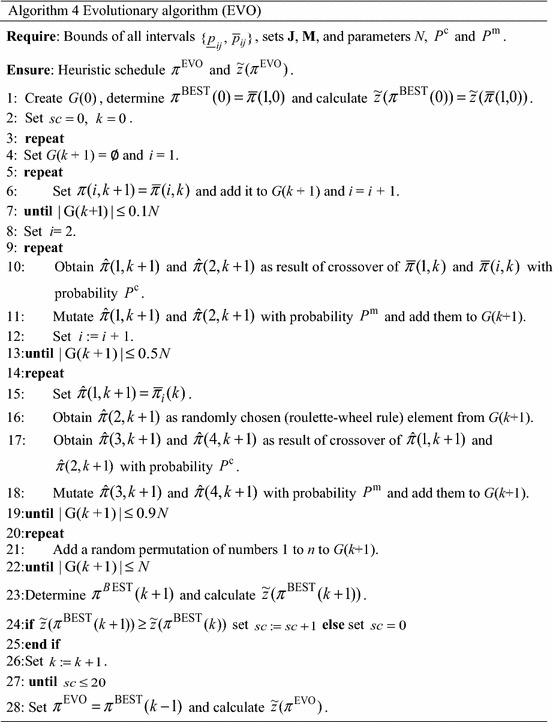



The permutation $\pi$ as a sequence of integer numbers from 1 to *n* is directly used as a chromosome, as well as $\tilde{z}$ serves as the fitness function. The standard ordered-crossover operator (OX) has been applied. The mutation consists of choosing randomly two positions in the permutation and swapping numbers occupying both positions. The full description of EVO can be found in Ćwik and Józefczyk (2015). Now, two improvements have been proposed in comparison with the presented there version. First of all, the method of generating new populations has been redesigned to provide more random populations avoiding too fast convergence to local minima.

The initial population $G(0)=\{\pi (1,0),\pi (2,0),\ldots ,\pi (N,0)\}$ consisting of *N* elements has the diverse structure. Namely, 90% of its elements are randomly generated according to the uniform distribution. The permutation $\pi ^{\mathrm{MIH}}$ determined by the MIH heuristic as well as its different mutations constitute the rest of *G*(0). The operator of mutation being the part of described evolutionary algorithm has been used to generate the mutated permutations of $\pi ^{\mathrm{MIH}}$. The generation of current population $G(k+1)=\{\pi (1,k+1),\pi (2,k+1),\ldots ,\pi (N,k+1)\}$, on the basis of the previous one *G*(*k*) has a more complex structure, where index *k* is incremented from 0 until the stop condition is fulfilled. The latter population is firstly evaluated according to the fitness function and sorted in the non-decreasing order to have a sequence $\bar{{G}}(k)=(\bar{{\pi }}(1,k),\bar{{\pi }}(2,k),\ldots ,\bar{{\pi }}(N,k))$ where $\tilde{z}(\bar{{\pi }}(l,k))\le \tilde{z}(\bar{{\pi }}(l+1,k)), \quad l=1,2,\ldots ,N-1$. The best 10% of such ordered elements (feasible solutions) are transferred to the current population $G(k+1)$ without any changes. After that, the best solution $\bar{{\pi }}(1,k)$ is crossed with subsequent solutions from $\bar{{G}}(k)$. The resulted offsprings undergo the mutation and are added to the created population. This combined process of crossover and mutation is repeated until 50% of the population size *N* is obtained. The next 40% of population elements are formed as results of the mutation which follows the crossover between the best solution $\bar{{\pi }}(1,k)$ and a solution randomly found in *G*(*k*) by the roulette-wheel selection mechanism. The remaining 10% of $G(k+1)$ is the outcome of a random generation of feasible solutions, which is to prevent too soon convergence of the algorithm to a local optimum. Moreover, the stop condition has been changed, i.e. the number of iterations without improvement *sc* has been changed from 5 to 20. The value of the best current solution $\pi ^{\mathrm{BEST}}(k+1)$ is calculated for every population (the iteration of the algorithm). If no improvement is observed in twenty consecutive iterations, the algorithm terminates and the best solution of the last iteration is returned as the final heuristic solution $\pi ^{\mathrm{EVO}}$ together with $\tilde{z}(\pi ^{\mathrm{EVO}})$.

As the result of performed tuning, the values of the algorithm’s parameters have been determined as: the size of population $N=60$, the probability of crossover $P^{\mathrm{c}}=0.95$ and the probability of mutation $P^{\mathrm{m}}=0.05$.

## Computational results

This section covers the algorithms’ experimental evaluation and their statistical analysis. All computations have been performed using a PC with Intel Core i5 CPU processor of 2.53 GHz with 4GB of RAM.

### Generation of problem instances

For the deterministic flow shop, there are in the literature known benchmark problems (Taillard 1993; Demirkol et al. 1998). It is admittedly possible to generalize those known problems to represent the case of interval processing times, e.g. by combining two problems separately for lower and upper bounds of the intervals. However, it would not be good benchmarks for the considered uncertain flow-shop as it would be impossible to have the values of maximum regret. Consequently, there are no known benchmarks available in the literature for the minmax regret permutation flow shop problem with interval processing times. So, we propose to generate random instances driven by integer numbers *C* and *K*. The lower and upper bounds of intervals are randomly chosen according to the discrete uniform distribution from intervals [1, *K*] and $[\underline{p}_{ij} ,\underline{p}_{ij} +C]$, respectively where $\underline{p}_{ij}$ is the result of the first generation. Hence, $\underline{p}_{ij} \in [1,K]$ and $\bar{{p}}_{ij} \in [\underline{p}_{ij} ,\underline{p}_{ij} +C]$. The ratio *C* / *K* represents the degree of uncertainty as the less is its value the less is the uncertainty’s significance. The above method is applied to all uncertainty intervals of the problem, thus, to obtain a new random problem instance, four parameters are required; *n*, *m*, *K*, and *C*. Whenever a random problem instance is referred to, it is denoted by a 4-tuple in the form {*n, m, K, C*}. If any element of the tuple needs to be randomized, it is generated according to the discrete uniform distribution from a set of integers denoted in braces or from an interval of integers denoted in brackets. For example, {20, {3,5}, 100, [10,100]} describes an instance with 20 jobs, random number of machines from the set {3,5} where $K=100$ and *C* is randomly selected integer from the interval [10,100].

### Experimental comparison and evaluation of the algorithms

In order to compare the algorithms over quality of generated solutions, performance indices are used for EVO and MIH algorithms

10 $$\begin{aligned} \delta ^{\mathrm{EVO}}=\frac{\tilde{z}(\pi ^{\mathrm{EVO}})}{\tilde{z}(\pi ^{\mathrm{CVE}})}, \quad \delta ^{\mathrm{MIH}}=\frac{\tilde{z}(\pi ^{\mathrm{MIH}})}{\tilde{z}(\pi ^{\mathrm{CVE}})}. \end{aligned}$$

**Fig. 1:**
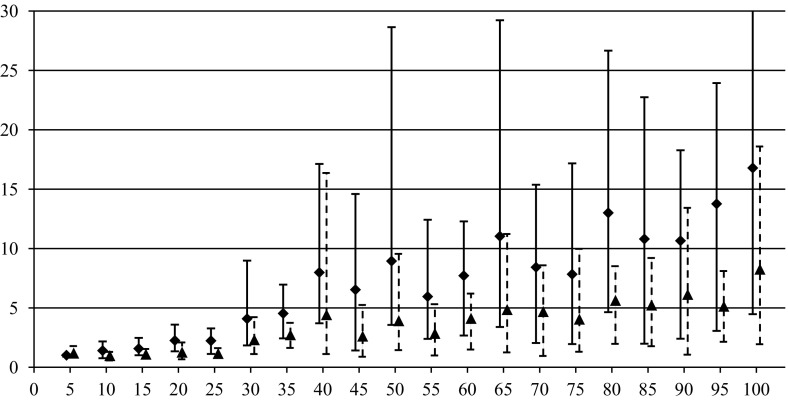
Dependence of $\delta _{\mathrm{avg}}^{\mathrm{EVO}}, \delta _{\min }^{\mathrm{EVO}} , \delta _{\max }^{\mathrm{EVO}}$ (*triangles*) and $\delta _{\mathrm{avg}}^{\mathrm{MIH}}, \delta _{\min }^{\mathrm{MIH}}, \delta _{\max }^{\mathrm{MIH}}$ (*squares*) on *n* for $m = 3$

**Fig. 2:**
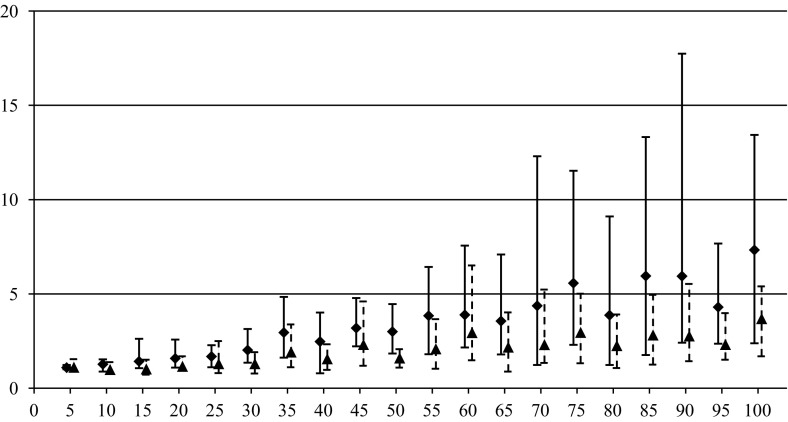
Dependence of $\delta _{\mathrm{avg}}^{\mathrm{EVO}} \delta _{\min }^{\mathrm{EVO}}, \delta _{\max }^{\mathrm{EVO}}$ (*triangles*) and $\delta _{\mathrm{avg}}^{\mathrm{MIH}}, \delta _{\min }^{\mathrm{MIH}}, \delta _{\max }^{\mathrm{MIH}}$ (*squares*) on *n* for $m=4$

**Fig. 3:**
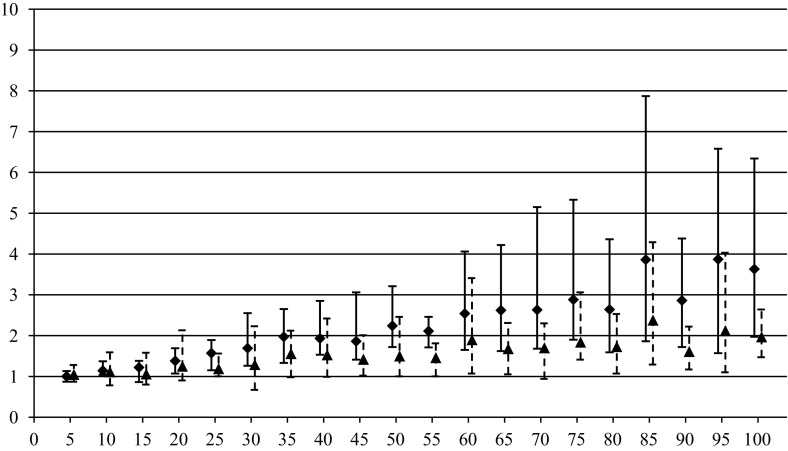
Dependence of $\delta _{\mathrm{avg}}^{\mathrm{EVO}}, \delta _{\min }^{\mathrm{EVO}}, \delta _{\max }^{\mathrm{EVO}}$ (*triangles*) and $\delta _{\mathrm{avg}}^{\mathrm{MIH}}, \delta _{\min }^{\mathrm{MIH}}, \delta _{\max }^{\mathrm{MIH}}$ (*squares*) on *n* for $m=5$

The values of performance indices express the relative disadvantage of EVO and MIH with respect to CVE. The greater are values of $\delta ^{\mathrm{EVO}}$ and $\delta ^{\mathrm{MIH}}$ the better is CVE. The algorithms have been compared for instances {*n*, *m*, 100, 50} where $m\in \left\{ {3,4,5} \right\}$ and $n\in \{5,10,15,\ldots ,100\}$. Ten independent random instances have been generated for each {*n*, *m*, 100, 50}, and corresponding values of both performance indices (10) have been calculated. The results for different *m* in the form of average $\delta _{\mathrm{avg}}^{(\cdot )}$, minimum $\delta _{\min }^{(\cdot )}$, and maximum $\delta _{\max }^{(\cdot )}$ values are presented in Tables 1, 2, and 3 as well as in Figs. 1, 2, and 3 by markers, bars of lower whiskers and bars of upper whiskers, respectively.

**Table 1:** Values of performance indices (10) for different *n* and $m=3$

*n*	$\delta _{\min }^{\mathrm{EVO}}$	$\delta _{\mathrm{avg}}^{\mathrm{EVO}}$	$\delta _{\max }^{\mathrm{EVO}}$	$\delta _{\min }^{\mathrm{MIH}}$	$\delta _{\mathrm{avg}}^{\mathrm{MIH}}$	$\delta _{\max }^{\mathrm{MIH}}$
5	0.89	1.19	1.78	0.89	1.01	1.08
10	0.57	0.98	1.29	0.76	1.4	2.17
15	0.77	1.1	1.54	1.01	1.57	2.47
20	0.67	1.27	2.09	1.34	2.25	3.59
25	0.85	1.16	1.61	1.12	2.23	3.27
30	1.11	2.32	4.22	1.84	4.08	8.98
35	1.63	2.74	3.74	2.43	4.53	6.96
40	1.11	4.43	16.36	3.7	7.98	17.12
45	0.88	2.62	5.24	1.41	6.53	14.59
50	1.44	3.92	9.55	3.56	8.94	28.64
55	0.99	2.85	5.31	2.39	5.95	12.42
60	1.49	4.12	6.2	2.67	7.71	12.28
65	1.25	4.86	11.22	3.39	11.03	29.22
70	0.95	4.69	8.58	2.04	8.42	15.37
75	1.3	4.05	9.97	1.95	7.83	17.17
80	1.97	5.63	8.51	4.64	13	26.66
85	1.77	5.24	9.2	1.98	10.8	22.74
90	1.06	6.14	13.42	2.4	10.65	18.27
95	2.14	5.14	8.1	3.06	13.76	23.93
100	1.94	8.26	18.59	4.47	16.78	55.59

**Table 2:** Values of performance indices (10) for different *n* and $m=4$

*n*	$\delta _{\mathrm{min}}^{\mathrm{EVO}}$	$\delta _{\mathrm{avg}}^{\mathrm{EVO}}$	$\delta _{\mathrm{max}}^{\mathrm{EVO}}$	$\delta _{\min }^{\mathrm{MIH}}$	$\delta _{\mathrm{avg}}^{\mathrm{MIH}}$	$\delta _{\max }^{\mathrm{MIH}}$
5	0.93	1.11	1.54	1	1.09	1.23
10	0.82	0.99	1.38	0.88	1.27	1.53
15	0.72	1.02	1.51	1.06	1.42	2.62
20	0.93	1.16	1.69	1.09	1.58	2.58
25	0.8	1.3	2.5	1.11	1.68	2.28
30	0.78	1.3	1.91	1.35	2.02	3.14
35	1.11	1.92	3.38	1.62	2.95	4.84
40	0.97	1.56	2.33	0.79	2.47	4.01
45	1.19	2.32	4.6	2.22	3.19	4.78
50	1.09	1.6	2.07	1.84	3	4.46
55	1.03	2.09	3.66	1.8	3.84	6.43
60	1.48	2.95	6.51	2.16	3.89	7.56
65	0.88	2.18	4.02	1.79	3.56	7.09
70	1.34	2.31	5.23	1.23	4.37	12.3
75	1.32	2.96	5.01	2.3	5.57	11.53
80	1.07	2.26	3.91	1.23	3.87	9.11
85	1.25	2.82	4.95	1.76	5.95	13.32
90	1.43	2.77	5.53	2.41	5.94	17.74
95	1.51	2.32	3.98	2.36	4.3	7.67
100	1.69	3.69	5.4	2.38	7.33	13.43

**Table 3:** Values of performance indices (10) for different *n* and $m=5$

*n*	$\delta _{\min }^{\mathrm{EVO}}$	$\delta _{\mathrm{avg}}^{\mathrm{EVO}}$	$\delta _{\mathrm{max}}^{\mathrm{EVO}}$	$\delta _{\min }^{\mathrm{MIH}}$	$\delta _{\mathrm{avg}}^{\mathrm{MIH}}$	$\delta _{\max }^{\mathrm{MIH}}$
5	0.87	1.05	1.28	0.87	1	1.13
10	0.78	1.11	1.59	1.04	1.14	1.37
15	0.8	1.06	1.58	0.86	1.22	1.38
20	0.9	1.25	2.13	1.07	1.38	1.69
25	1	1.19	1.56	1.15	1.57	1.89
30	0.67	1.29	2.23	1.26	1.69	2.55
35	0.98	1.56	2.12	1.33	1.97	2.65
40	0.99	1.53	2.42	1.53	1.93	2.85
45	1.02	1.42	2.01	1.41	1.86	3.06
50	1	1.5	2.46	1.72	2.24	3.21
55	1	1.46	1.81	1.71	2.11	2.46
60	1.07	1.9	3.41	1.65	2.54	4.06
65	1.05	1.68	2.31	1.62	2.62	4.22
70	0.94	1.7	2.3	1.68	2.63	5.15
75	1.41	1.84	3.06	1.9	2.88	5.33
80	1.07	1.73	2.53	1.59	2.64	4.36
85	1.29	2.38	4.29	1.86	3.86	7.87
90	1.17	1.61	2.22	1.72	2.86	4.38
95	1.1	2.12	4.03	1.57	3.87	6.58
100	1.47	1.97	2.64	1.97	3.63	6.34

This experiment confirmed the supremacy of CVE which can return better results than EVO and MIH in average up to respectively 8 and 16 times ($m =3, n =100$). The advantage of CVE increases with the growth of *n* for all *m*, however, the difference between the algorithms is the most noticeable for $m = 3$, and it decreases for the greater values of *m*. There are only two instances, i.e. $m = 3, n = 10$ and $m = 4, n = 10$ where EVO slightly outperforms CVE. MIH turned out absolutely the worst on the quality.

**Fig. 4:**
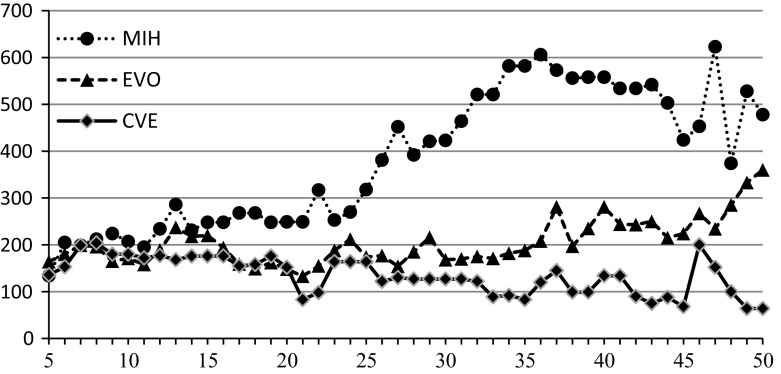
Dependence of $\tilde{z}$ on *n* for $m=3$

**Fig. 5:**
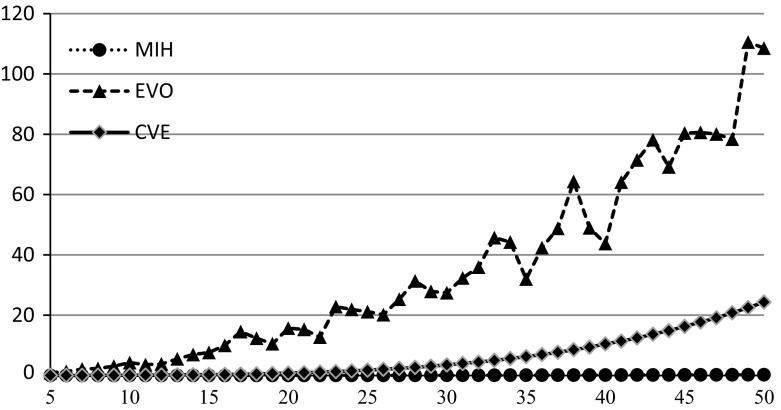
Dependence of *T* on *n* for $m=3$

The second experiment has been conducted to make the experimental evaluation of the algorithms more versatile. Now, a single randomly generated instance {50, 3, 100, 5} is the basis for all performed calculations, unlike the previous case when every instance was generated independently. The sub-instance {*n*, 3, 100, 5} was created by taking numerical data from fixed {50, 3, 100, 5} for $n=5,6,\ldots ,50$. All the algorithms have been launched for such sub-instances, and both values of $\tilde{z}$ and computational times *T* in seconds have been returned. The algorithm EVO has been executed five times due to its probabilistic operation and averaged values are presented. The results are given in Table 4 and Figs. 4 and 5.

The results confirm the previous observation that CVE is undoubtedly better than EVO regarding $\tilde{z}$ for bigger $n (n>20)$. The difference is not visible for $n<20$. CVE is also better than MIH. On the other hand, CVE cannot compete with MIH regarding the computational time *T*, but it needs substantially less time than EVO. It takes less than 24 s. for $n=50$ that seems to be a good result.

**Fig. 6:**
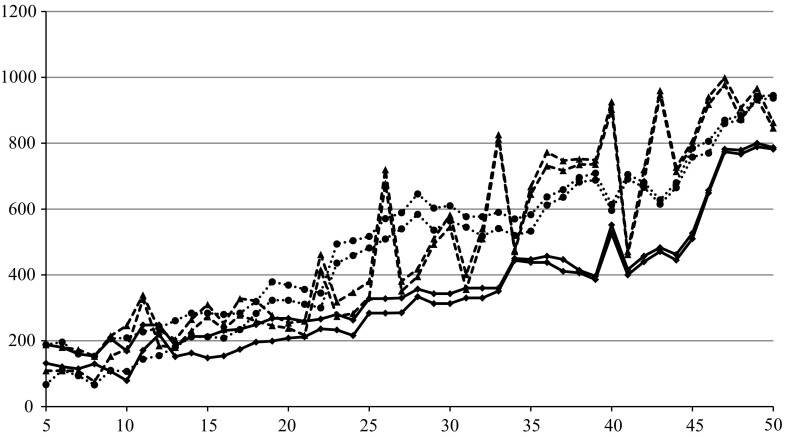
Dependence of $z_{\mathrm{LB}} (\pi ^{(\cdot )})$ and $z_{\mathrm{UB}} (\pi ^{(\cdot )})$ on *n* for MIH (*dots*), EVO (*triangles*) and CVE (*squares*)

The similar experiment has been performed for another random instance {50, 3, 100,50} and its resulting sub-instances. The objective of this experiment was to learn about the estimated relation among values of (3) for schedules obtained by all three algorithms. As the precise calculation of (3) is not possible due to its complexity, its lower bound $z_{\mathrm{LB}} (\pi )$ and upper bound $z_{\mathrm{UB}} (\pi )$ can be determined, where:

11 $$\begin{aligned} z_{\mathrm{LB}} (\pi )= & {} \mathop {\max }\limits _{p\in p} \left( {C_{\max } (\pi ,p)-C_{\max } (\pi ^{\mathrm{NEH}(p)},p)} \right) , \end{aligned}$$

12 $$\begin{aligned} z_{\mathrm{UB}} (\pi )= & {} \mathop {\max }\limits _{p\in p} \left( {C_{\max } (\pi ,p)-{C}'_{\max , \mathrm{LB}} (p)} \right) , \end{aligned}$$

and $\pi ^{\mathrm{NEH}(p)}$, is calculated by NEH for fixed *p* while ${C}'_{\max , \mathrm{LB}} (p)$ is given by (4). The values presented in Table 5 and depicted in Fig. 6 enable us for more exact evaluation of these three schedules as the relaxation of SP2 is not applied, and the worst-case scenarios have been directly calculated. The real value of (3) always lies in the belts bounded by $z_{\mathrm{LB}} (\pi )$ and $z_{\mathrm{UB}} (\pi )$. The locations of belts indicate the previous conclusion about the advantage of CVE with respect to EVO and MIH and the supremacy of CVE.

To sum up, the experiments indicated the relationship among all three algorithms with respect to $\tilde{z}$; CVE turned out to be the best algorithm for the essential majority of checked instances. The execution times additionally asserted the usefulness of CVE which cannot compete with MIH, but it is substantially faster than EVO algorithm especially for the greater values of *n*. All experiments showed the supremacy of CVE with respect to $\tilde{z}$ less evident for small values of *n* or high values of *m*. In the next sub-section, this observation is undergone a more comprehensive statistical analysis to avoid the possible interpretation on its accidental character.

**Fig. 7:**
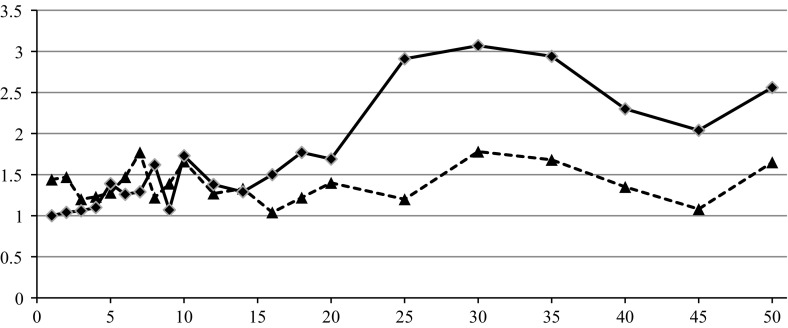
Dependence of $\delta _{\mathrm{avg}}^{(\cdot )}$ (EVO, *triangles*, MIH, *squares*) on *C* for $n=20, m=3\;\hbox {and}\;K=100$

The last experiment that has been conducted aimed to verify how the algorithms’ quality behaves for less uncertain data that is for smaller values of ratio *C*/*K*. The instances {20, 3, 100, *C*} have been taken into account. Ten independent instances of the problem have been randomly generated for every value of *C* from the set $\{1,2,3,4,5,6,7,8,9,10,12,14,16,18,20,25,30,35,40,45,50\}$. Each instance has been solved with all three algorithms, and averaged values of (10) have been calculated. The results are presented in Fig. 7.

No significant influence of *C* on $\delta _{\mathrm{avg}}^{\mathrm{EVO}}$ and $\delta _{\mathrm{avg}}^{\mathrm{MIH}}$ has been observed for $C>20$. Then, CVE outperforms EVO and MIH. For smaller values of $C, \delta ^{\mathrm{MIH}}$ converges towards 1. This observation has been expected because decreasing of the level of problem uncertainty implies the larger similarity between algorithms CVE and MIH.

### Statistical analysis

The results of performed experiments enable us to put forward a hypothesis on the comparison of algorithms in terms of the criterion (5). The following inequalities hold for the majority part of experiments

13 $$\begin{aligned} \tilde{z}\left( \pi ^{\mathrm{CVE}}\right)<\tilde{z}\left( \pi ^{\mathrm{EVO}}\right) <\tilde{z}\left( \pi ^{\mathrm{MIH}}\right) . \end{aligned}$$

**Table 4:** Values of $\tilde{z}$ and computational time *T* for $m=3$ and different *n*

*n*	$\tilde{z}(\pi ^{\mathrm{MIH}})$	$T(\pi ^{\mathrm{MIH}})$	$\tilde{z}(\pi ^{\mathrm{EVO}})$	$T(\pi ^{\mathrm{EVO}})$	$\tilde{z}(\pi ^{\mathrm{CVE}})$	$T(\pi ^{\mathrm{CVE}})$
5	134	<0.01	164	0.90	136	0.01
6	205	<0.01	180	1.34	153	0.01
7	199	<0.01	199	2.13	199	0.02
8	212	<0.01	196	2.32	204	0.04
9	224	<0.01	165	3.09	180	0.05
10	207	<0.01	171	4.25	180	0.07
11	195	<0.01	158	3.58	172	0.10
12	234	<0.01	189	3.82	177	0.12
13	286	0.01	237	5.51	168	0.17
14	231	0.01	218	6.93	176	0.21
15	248	0.01	220	7.63	176	0.27
16	248	0.01	194	9.96	176	0.34
17	268	0.01	159	14.54	155	0.42
18	268	0.02	149	12.34	158	0.52
19	248	0.02	162	10.52	176	0.67
20	249	0.02	148	15.70	152	0.78
21	249	0.02	133	15.29	83	0.93
22	317	0.03	155	12.70	98	1.12
23	253	0.03	188	22.83	164	1.30
24	270	0.03	212	21.91	164	1.52
25	318	0.04	174	21.15	164	1.77
26	381	0.04	177	20.15	122	2.05
27	452	0.04	154	25.27	130	2.35
28	392	0.05	185	31.34	127	2.70
29	421	0.05	216	27.87	127	3.07
30	423	0.06	168	27.42	127	3.50
31	464	0.06	170	32.32	127	3.97
32	521	0.07	175	35.93	122	4.43
33	521	0.08	171	45.70	89	5.01
34	582	0.10	182	44.24	92	5.61
35	582	0.10	188	31.97	83	6.25
36	606	0.10	208	42.37	120	6.94
37	573	0.11	281	48.78	145	7.68
38	556	0.12	197	64.34	99	8.54
39	558	0.13	235	48.94	99	9.37
40	558	0.14	281	43.75	134	10.45
41	534	0.15	244	64.10	134	11.38
42	534	0.16	243	71.47	90	12.45
43	542	0.17	250	78.16	75	13.67
44	503	0.18	215	69.15	88	14.85
45	424	0.19	224	80.39	68	16.26
46	453	0.21	267	80.62	200	17.72
47	623	0.21	234	80.06	152	19.10
48	374	0.23	285	78.37	100	20.78
49	528	0.24	333	110.55	64	22.48
50	478	0.25	360	108.57	64	24.32

To show that there is a significant statistical difference among the algorithms independent of the problem size or *C* / *K* ratio, we will address both inequalities separately with the Wilcoxon paired-rank test (Wilcoxon 1945; Derrac et al. 2011). Three hundred independent samples have been generated according to the 4-tuple {{5, 50}, {3, 5}, 100, {10, 100}}, and the calculated values of $\tilde{z}(\pi ^{\mathrm{CVE}}), \tilde{z}(\pi ^{\mathrm{EVO}})$ and $\tilde{z}(\pi ^{\mathrm{MIH}})$ have been the basis of two statistical tests comparing $\tilde{z}(\pi ^{\mathrm{MIH}}), \tilde{z}(\pi ^{\mathrm{EVO}})$ and $\tilde{z}(\pi ^{\mathrm{EVO}}), \tilde{z}(\pi ^{\mathrm{CVE}})$.

**Table 5:** Values of lower and upper bounds of *z* given in (9) obtained by MIH, EVO and CVE

n	$z_{\mathrm{LB}} (\pi ^{\mathrm{MIH}})$	$z_{\mathrm{UB}} (\pi ^{\mathrm{MIH}})$	$z_{\mathrm{LB}} (\pi ^{\mathrm{EVO}})$	$z_{\mathrm{UB}} (\pi ^{\mathrm{EVO}})$	$z_{\mathrm{LB}} (\pi ^{\mathrm{CVE}})$	$z_{\mathrm{UB}} (\pi ^{\mathrm{CVE}})$
5	67	188	109	188	132	188
6	110	196	109	180	121	180
7	94	160	108	173	115	160
8	66	153	76	153	130	153
9	110	206	153	215	107	206
10	107	209	175	246	79	169
11	144	227	326	339	171	248
12	155	239	185	234	219	248
13	181	261	180	203	152	185
14	212	284	230	265	163	213
15	212	284	273	310	148	213
16	208	280	235	252	154	231
17	234	284	278	328	174	235
18	283	319	259	321	196	249
19	323	379	246	277	199	268
20	323	369	238	254	208	268
21	311	356	217	260	212	259
22	300	345	415	462	236	266
23	436	494	274	318	233	280
24	459	504	283	347	216	263
25	482	517	333	380	284	328
26	509	571	678	720	284	328
27	540	589	351	381	285	330
28	584	646	395	418	334	357
29	536	603	493	510	313	343
30	564	610	545	582	313	343
31	545	577	356	401	330	360
32	518	577	510	540	330	360
33	541	590	813	826	351	360
34	520	570	471	476	444	450
35	533	583	645	668	438	447
36	612	637	731	773	438	457
37	636	659	717	747	411	447
38	682	696	735	752	406	414
39	688	709	736	749	386	396
40	596	614	903	926	526	552
41	691	705	462	471	400	416
42	666	681	685	720	440	457
43	615	628	947	960	471	483
44	665	680	714	727	445	462
45	758	785	794	807	510	527
46	770	806	918	941	649	657
47	860	870	978	999	774	782
48	871	884	873	907	767	779
49	932	943	935	967	789	800
50	937	945	846	863	782	787

To perform the Wilcoxon test for $\tilde{z}(\pi ^{\mathrm{MIH}})$ and $\tilde{z}(\pi ^{\mathrm{EVO}})$, the null-hypothesis has been defined as:

$H_0$ : There is no statistically significant difference between the values of $\tilde{z}(\pi ^{\mathrm{MIH}})$ and $\tilde{z}(\pi ^{\mathrm{EVO}})$ with the alternative hypothesis $H_1 : \tilde{z}(\pi ^{\mathrm{MIH}})\ge \tilde{z}(\pi ^{\mathrm{EVO}})$.

To verify the hypothesis, the differences $\tilde{z}(\pi ^{\mathrm{MIH}})-\tilde{z}(\pi ^{\mathrm{EVO}})$ for each pair of 300 instances have been calculated, and 295 of them have been remained after discarding all zero-differences. Then, the remaining differences’ absolute values have been ranked, and the signed rank has been determined based on the sign of the difference. All signed ranks have been added up to obtain the statistic $W=37794.5$. It is known that for large sample size, if the compared random variables have the same distributions than *W* tends to the normal distribution with the mean value $\mu _\mathrm{W} =0$ and a standard deviation equal to: $\sigma _\mathrm{W} =\sqrt{\frac{N_\mathrm{W} (N_\mathrm{W} +1)(2N_\mathrm{W} +1)}{6}}=2932.75$ for $N_\mathrm{W} =295$.

Finally, $z_\mathrm{W}$ has been calculated after adding to *W* the value -0.5 as the correction for continuity $z_\mathrm{W} =\frac{(W-\mu _\mathrm{W} )-0.5}{\sigma _\mathrm{W} }= 12.88$.

It outperforms the critical value $z_{\mathrm{one}{\text {-}}\mathrm{tailed}}^{0.0005} = 3.291$ valid for the one-tailed test and the significance level $\alpha =0.0005$. Therefore, we can conclude that there is a strong statistical evidence of rejecting the null-hypothesis which confirms right-hand part of inequality (11).

The same test has been performed to compare EVO and CVE algorithms with the analogous hypotheses $$H_0 $$ and $$H_1 $$. The numerical results are as follows: $$W = 3205319, \mu _\mathrm{W} =0, \sigma _\mathrm{W} =\sqrt{\frac{N_\mathrm{W} (N_\mathrm{W} +1)(2N_\mathrm{W} +1)}{6}}=2726.82$$ for $$N_\mathrm{W} =281$$ after discarding 19 zero-differences, $$z_\mathrm{W} =\frac{(W-\mu _\mathrm{W} )-0.5}{\sigma _\mathrm{W} }=11.75$$. Like for the previous test, there is very strong evidence to reject the null hypothesis, which confirms the left-hand inequality in (11).

## Conclusions

The minmax regret version of the permutation flow-shop with the number of machines greater than two, unlimited buffers, interval processing times and the makespan as a criterion has been investigated. The paper extends previous works on the difficult problem with many machines when the deterministic counterpart is NP-hard. The constructive algorithm CVE has been introduced for the first time and experimentally evaluated with respect to two other heuristic algorithms: the new version of the previously elaborated evolutionary algorithm (EVO) and the evident middle interval algorithm (MIH). The value of maximum regret, as well as the computational time has been the basis for the comparison. The new algorithm CVE substantially outperforms EVO and MIH for both bases of the comparison. It turned out that CVE not only is faster than EVO, but it also is substantially better in terms of the value of minmax regret (5), and, consequently, it is recommended for real-world applications.

The elaboration of a branch and bound based exact algorithm is now in progress to have the more credible basis for the evaluation of heuristic algorithms. Moreover, searching for new more efficient heuristic approaches for the considered in the paper minmax regret flow-shop with interval processing times is still under development. It mainly concerns the approximation of the criterion value which is itself an NP-hard issue. The usefulness of other heuristics for the flow-shop like CDS (Campbell, Dudek and Smith) will also be verified (Pinedo 2008). The idea of elaborating of time-effective heuristics for different interval minmax regret combinatorial optimization problems will be continued primarily for task scheduling problems with the open-shop as the first example.
